# Educational Differences in Diabetes Mortality among Hispanics in the United States: An Epidemiological Analysis of Vital Statistics Data (1989–2018)

**DOI:** 10.3390/jcm10194498

**Published:** 2021-09-29

**Authors:** Alberto Barcelo, Alfredo Valdivia, Angelo Sabag, Juan Pablo Rey-Lopez, Arise Garcia de Siqueira Galil, Fernando A.B. Colugnati, María Pastor-Valero

**Affiliations:** 1 Miller School of Medicine, Department of Public Health Science, University of Miami, Miami, FL 33136, USA; axv631@med.miami.edu; 2 Departamento de Clinica Medica, Universidade Federal de Juiz de Fora, Juiz de Fora 36036-330, Brazil; 3 NICM Health Research Institute, Western Sydney University, Westmead, NSW 2145, Australia; A.Sabag@westernsydney.edu.au; 4 Faculty of Health Sciences, International University of Valencia, 46003 Valencia, Spain; jpreylopez@hotmail.com; 5 Faculty of Sport, Universidad Católica San Antonio de Murcia, 30107 Murcia, Spain; 6 Departamento de Internato, Facultade de Medicina, Universidade Federal de Juiz de Fora, Juiz de Fora 36036-330, Brazil; galilarise@gmail.com; 7 Department of Post-Graduation, School of Medicine, Universidade Federal de Juiz de Fora, Juiz de Fora 36036-330, Brazil; fernando.colugnati@medicina.ufjf.br; 8 Departamento de Salud Pública, História de la Ciencia y Ginecología, Universidad Miguel Hernández de Elche, 03550 Sant Joan d’Alacant, Spain; mpastor@umh.es; 9 Centro de Investigación Biomédica en Red de Epidemiología y Salud Pública (CIBERESP), 28029 Madrid, Spain

**Keywords:** USA Hispanics, diabetes mortality, education, inequalities

## Abstract

Background: Diabetes accounted for approximately 10% of all-cause mortality among those 20–79 years of age worldwide in 2019. In 1986–1989, Hispanics in the United States of America (USA) represented 6.9% of the national population with diabetes, and this proportion increased to 15.1% in 2010–2014. Recently published findings demonstrated the impact of attained education on amenable mortality attributable to diabetes among Non-Hispanic Whites (NHWs) and Non-Hispanic Blacks (HNBs). Previous cohort studies have shown that low education is also a detrimental factor for diabetes mortality among the Hispanic population in the USA. However, the long-term impact of low education on diabetes mortality among Hispanics in the USA is yet to be determined. Aims and methods: The aim of this study was to measure the impact of achieving a 12th-grade education on amenable mortality due to diabetes among Hispanics in the USA from 1989 to 2018. We used a time-series designed to analyze death certificate data of Hispanic-classified men and women, aged 25 to 74 years, whose underlying cause of death was diabetes, between 1989 and 2018. Death certificate data from the USA National Center for Health Statistics was downloaded, as well as USA population estimates by age, sex, and ethnicity from the USA Census Bureau. The analyses were undertaken using JointPoint software and the Age–Period–Cohort Web Tool, both developed by the USA National Cancer Institute. Results: The analyses showed that between 1989 to 2018, age- and sex-standardized diabetes mortality rates among the least educated individuals were higher than those among the most educated individuals (both sexes together, *p* = 0.036; males, *p* = 0.053; females, *p* = 0.036). The difference between the least and most educated individuals became more pronounced in recent years, as shown by independent confidence intervals across the study period. Sex-based analyses revealed that the age-adjUSAted diabetes mortality rate had increased to a greater extent among the least educated males and females, respectively, than among the most educated. Conclusions: The results of the analyses demonstrated a powerful effect of low education on amenable mortality attributable to diabetes among the Hispanic population in the USA. As an increasing prevalence of diabetes among the least educated Hispanics has been reported, there is a great need to identify and implement effective preventive services, self-management, and quality care practices, that may assist in reducing the growing disparity among those most vulnerable, such as minority populations.

## 1. Introduction

In 2019, diabetes was estimated to account for approximately 10% of all-cause mortality among those 20–79 years of age worldwide [1]. Between 1986 and 1989, Hispanics represented 6.9% of the total population with diabetes in the United States (USA), and this proportion increased to 15.1% from 2010 to 2014 [2]. The prevalence of diabetes in Hispanics is higher than among Non-Hispanic Whites (NHWs) in the USA. According to the Center for Disease Control (CDC), between 2013 to 2016, approximately 14.7% of Hispanics had diabetes, whereas the prevalence of diabetes among NHWs during this same period was 11.9% [3,4]. Furthermore, the CDC predicts that over half of the total Hispanic adult population in the USA will develop type 2 diabetes in their lifetime [5]. These findings are compounded by other reports highlighting that the onset of diabetes tends to occur earlier among Hispanics than among NHWs for reasons such as genetic predisposition and lifestyle factors to NHWs [6,7,8]. Given the relationship between obesity and diabetes, it is unsurprising that the prevalence of obesity in 2015–2016 was higher among the Hispanic male population than among males from other race/ethnic groups in the USA [9]. In fact, Hispanic men ranked second, only behind NHB women [9], when comparing all gender-specific prevalence of obesity by race/ethnicity in the USA in 2015–2016. As highlighted earlier, lifestyle factors, including dietary practices, play a significant role in the increased prevalence of diabetes, and similar can be said with respect to obesity, as recent data has shown that approximately 50% of total caloric intake among Hispanics in the USA is derived from ultra-processed foods [10]. Furthermore, the increased prevalence of obesity may be exacerbated by cultural factors, such as perceptions of overweight and obesity, which have been shown to differ among various cultural and ethnic groups in the USA [6,7,8,11]. 

The mortality rate in the USA has been in a consistent decline from 1969 to 2013 [3]. Diabetes mortality has also decreased during this period [12], which is due to an array of factors including advancements in diabetes-related care [13]. However, many deaths, especially among the young, may still be susceptible to be further reduced by additional public health and policy actions. Amenable mortality is defined as deaths from diseases, including diabetes, that are potentially preventable, given the availability of effective and timely healthcare below a certain age, which they established for most diseases was 74 years of age [14]. The term was created in 1976 by Runstein [14] to quantify deaths that could have been avoided with appropriate healthcare. The term amenable was also used by Nolte et al. in most recent publications [15,16,17]. As such, amenable mortality may serve as an effective proxy of healthcare performance. While amenable mortality is impacted by various factors, educational attainment has been identified as a significant predictor of all-cause mortality in the USA [18,19,20]. Previous cohort studies have reported educational differences in general mortality among the USA population with diabetes, but this effect was not observed among Hispanics at the time [21,22]; a more recent cohort study reported a decline in general mortality among the least educated Hispanics with diabetes between 1997 to 2011 [23]. We previously reported that the age-adjusted mortality rates for the leading chronic disease, including diabetes, increased among the least educated, as compared to the most educated, and these differences were seen in both sexes among NHWs and NHBs between 1990 to 2015 [24]. While these recently published findings demonstrated the long-term impact of low education on diabetes and other chronic diseases mortality, the Hispanic population, who experience unique barriers to healthcare access and education in the USA, were not included. Therefore, the objective of this study was to measure the impact of achieving a 12th-grade education on amenable mortality attributable to diabetes among Hispanics in the United States from 1989 to 2018.

## 2. Material and Methods

### 2.1. Study Design and Population

The Hispanic or Latino population is classified as anyone from Cuban, Mexican, Puerto Rican, South or Central American, or another Spanish culture or origin, regardless of race, residing in the USA [25].

For our analysis, we used death certificate data, which included age, sex, and education, of Hispanic-classified men and women, aged 25 to 74 years, whose underlying cause of death was diabetes, between 1989 and 2018, downloaded from the USA National Center for Health Statistics Database [26], and the USA population estimates from the USA Census Bureau [27] by age, sex, and ethnicity, between 1989 to 2018, were downloaded to calculate age- and sex-adjusted diabetes mortality rates. The underlying cause of death according to ICD-9 (cause 250) was used for information recorded between 1989 and 1998 [28,29], and the ICD-10 list for the underlying cause of death was used for information recorded between 1999 and 2018 (causes E10–E14) [29,30]. As the analysis was undertaken using publicly available information, the ethical review of the study protocol was not required. 

### 2.2. Primary Outcome 

#### Amenable Mortality

The analysis was restricted to Hispanic individuals aged 25–74 years whose underlying cause of death was diabetes in the USA between 1989 and 2018. An early study by Nolte et al. limited the age of diabetes deaths to 49 years of age in their analyses due to the controversy regarding the preventability of diabetes deaths at older ages [17,31], however, as diabetes mortality in the USA has declined in recent years, this analysis included deaths up to 74 years to better reflect current patterns [12].

### 2.3. Stratifying Variables

Age-adjusted diabetes mortality rates were stratified by sex and education level. Data on attained education and ethnicity were extracted from the death certificate databases. The rationale for comparing educational attainment above or below the 12th-grade level is supported by existing literature showing that those who attain 12 years or more years of education are more likely to achieve a better standard of living, including higher income, as well as greater access to housing and health insurance [24,32,33]. Although it is possible that attaining higher education degrees may contribute to a longer life, a low number of subjects in some groups precluded any comparison for different categories of higher education.

### 2.4. Statistical Analysis

The primary analysis of this observational study involved assessing the annual percentage change in age-standardized mortality rates for Hispanic residents in the USA by education level (lower than 12th grade or 12th grade or more) between 1989 and 2018. The analyses were undertaken using the JointPoint Regression Program [34,35,36] and the Age–Period–Cohort Web Tool (APCWT), both developed by the USA National Cancer Institute, as previously described by Xie et al. [37]. Age-adjusted mortality rates were calculated using the USA 2000 Census population as the standard. The JointPoint program calculates trends and estimates the annual percent change (APC) assuming that rate changes are similar on a log scale for each consecutive period. The average annual percent change (AAPC) was computed as a summary measure of the trend over the whole period 1989–2018. The *p* values for pairwise comparisons were reported instead of thresholds (i.e., *p* < 0.05) for statistical significance, as recently suggested by Wasserstein [38], so the reader can look at probabilities as a continuous number. Although we preferred to compare estimates rather than probabilities, for some indicators, we displayed a 95% CI.

An age–period–cohort regression was also performed using the annual percent cohort change (APCC) to examine the effect of age, birth cohort, and year of death on age-adjusted mortality rates, using the APCWT. The APCWT provides a smoothed summary curves from age-specific mortality rates. The indicators called local drifts are generated from log-linear regressions. Technical information on modeling is readily available elsewhere [39,40]. The APCWT provides age-adjusted effects for age, period (year of death), and cohort (year of birth), capturing differences in risk over time and facilitating the interpretation of trends. The APC model selects the central age group, calendar year, and birth cohort as reference values to calculate age, calendar year, and cohort deviation of each sex and education group. These statistical methods were used to generate the data presented in some of the figures. The year 2002 was used as a reference year for the period effect, while 1954 was used as a reference year for the cohort effect and 50 years of age for the age effect. The midpoints were automatically selected as standard by ACPWT and are displayed at the bottom of each figure [39,40]. As the APCWT does not provide comparisons between groups, the differences between groups were determined by a visual appraisal of confidence intervals to determine whether they overlapped or not at any time point [39,40]. Data were analyzed using five-year intervals from 1989 to 2018.

## 3. Results

A total of 97,758 Hispanic deaths were included in the analysis. Males represented 54% of cases. The total number of diabetes deaths, in both males and females, increased steadily from 7698 in 1989–1993 to 24,603 in 2014–2018. Crude mortality rates were higher among those the least educated than the most educated, males and females, respectively, at any point in time. Mortality rates increased consistently with time among the least educated males and females, respectively. Mortality rates varied among the most educated males and females, respectively, during the same period (Table 1). At the end of the study period 2014–2018, crude mortality rates were higher for the least educated than for the most educated among both sexes, respectively.

**Figure 1:**
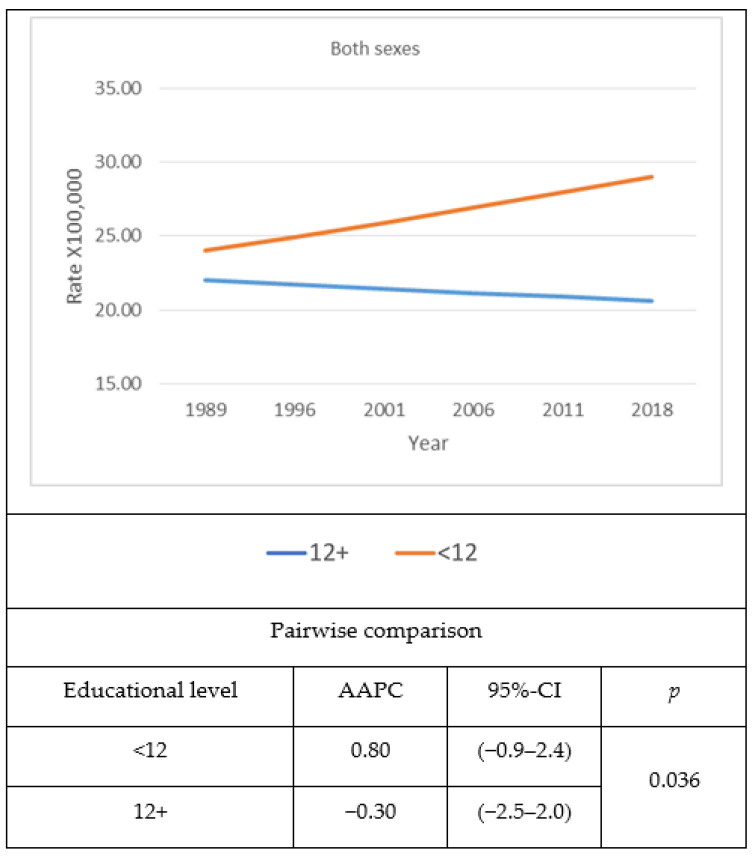
Time trend of age- and sex-adjusted diabetes mortality and average annual percent change (AAPC) among Hispanics in the USA by education level and year from 1989 to 2018. The 2000 USA Census Population was used as standard.

**Figure 2:**
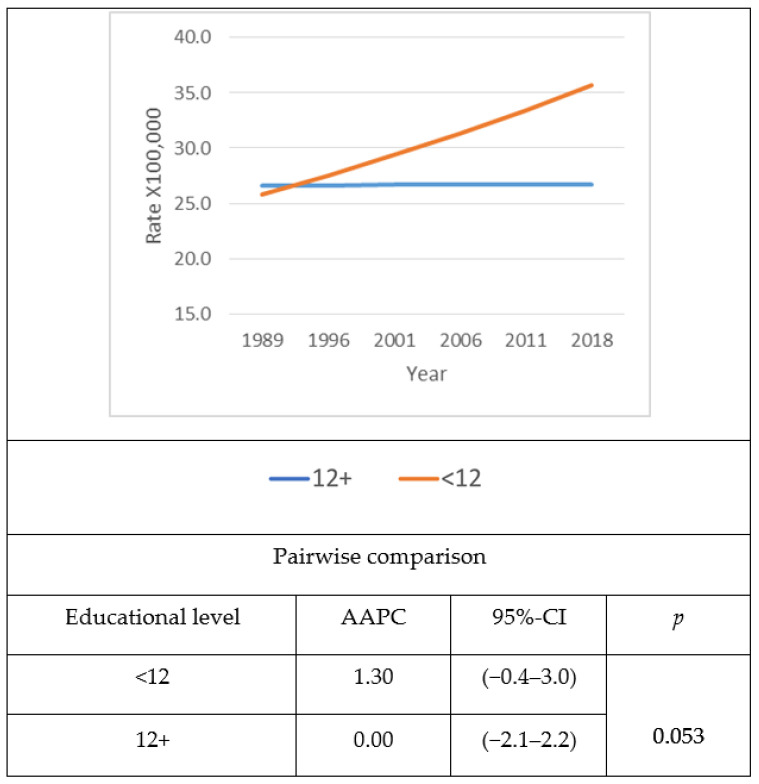
Time trend of age- and sex-adjusted diabetes mortality and average annual percent change (AAPC) among Hispanic males in the USA by education level and year from 1989 to 2018. The 2000 USA Census Population was used as standard.

**Figure 3:**
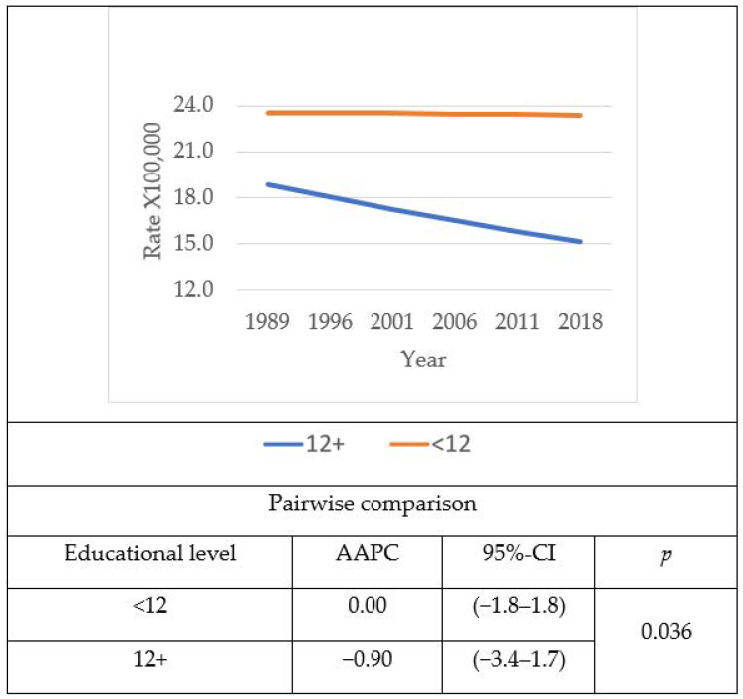
Time trend of age- and sex-adjusted diabetes mortality and average annual percent change (AAPC) among Hispanic females in the US by education level and year from 1989 to 2018. The 2000 USA Census Population was used as standard.

Analyses involving combined males and females’ age- and sex-adjusted diabetes mortality rates using the USA 2000 Census population as standard (Figure 1) showed that age- and sex-adjusted diabetes mortality rates decreased among the most educated, while they remained stable among the least educated. The difference in age- and sex-adjusted diabetes mortality between those with higher education, compared to those with lower education, widened with time, due to the divergent trend between the two groups. The AAPC for both sexes together increased (0.8%) among the least educated and decreased (−0.30%) among the most educated, resulting in a significant gap (*p* = 0.036) between the two educational groups. Sex-based analyses revealed that among males, age-standardized diabetes mortality rates for the least educated increased with time, while they remained stable among the most educated (Figure 2). The AAPC showed a slightly widening trend (*p* = 0.053) as a consequence of increasing age-adjusted mortality rates among the least educated (1.3%), while the age-adjusted mortality among the most educated males remained stable, with an AAPC demonstrating no variation during the time period (0.0%). However, the gap between the least and the most educated age-adjusted mortality rates for Hispanic males was considerably large, especially during the later years. Among females, age-adjusted mortality rates remained constantly high among the least educated during the time period, whereas age-adjusted mortality rates among the most educated were in continuous decline (Figure 3). There was a marked difference in AAPC (*p* = 0.036) when comparing the most educated, which decreased with time (−0.30%) to the least, which remained constant with time (0.0%).

**Figure 4:**
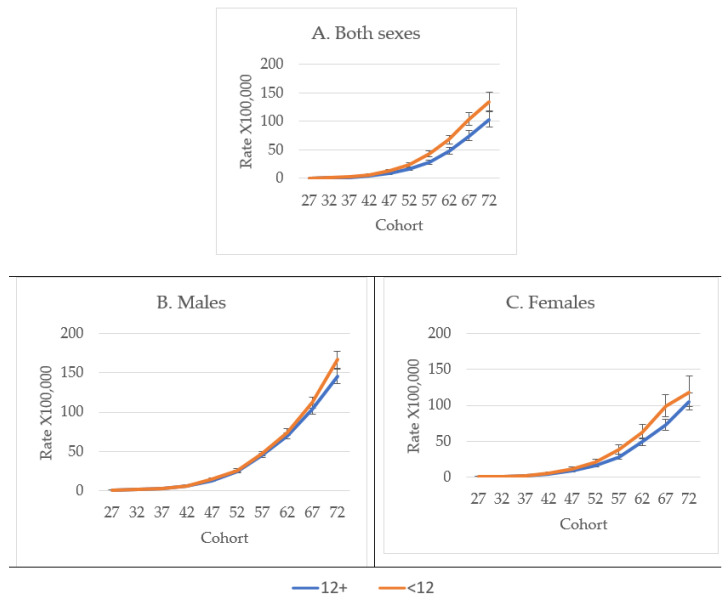
Age- and sex-adjusted diabetes mortality rate among Hispanics in the USA by age, sex, and education level from 1989 to 2018.

Age-adjusted mortality rates showed increasing trends with age among both sexes and educational levels. From 47 years of age, the least educated showed higher mortality rates than the most educated for analyses combining both sexes, as well as when stratified by males and females. The difference in mortality rates between the least and the most educated women progressively increased after the age of 47 years up to 67 years, when the difference between the most and least educated began to diminish (Figure 4A–C).

**Figure 5:**
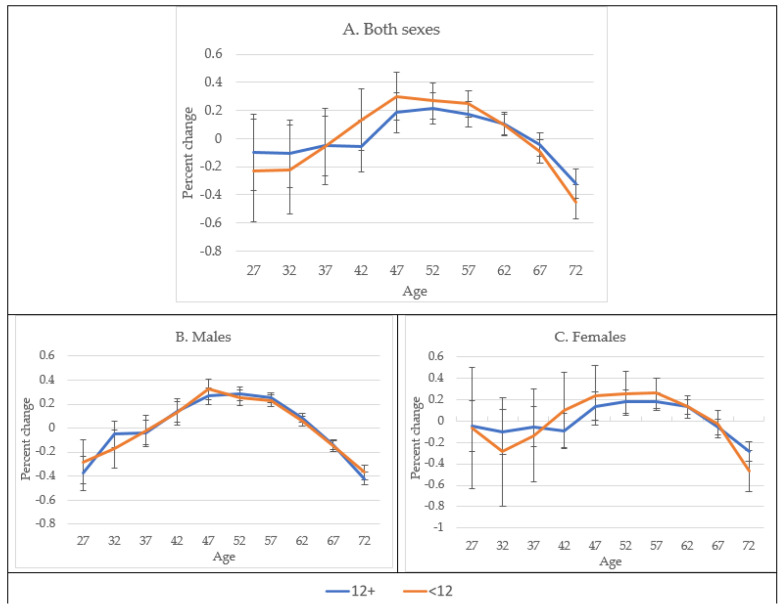
Age deviation of diabetes mortality rates (in percent) among Hispanics by sex and education level, United States, 1989–2018. Percent change was compared to mortality at age 50 years.

Age deviation, with respect to mortality at 50 years of age, of the age-specific diabetes mortality rates indicated higher mortality rates among the least educated than among the most educated, in both sexes together, between 37 and 57 years of age. Age-adjusted mortality rates varied with age in both sexes, among males, and among females. Age deviation peaked at 47 and 52 years of age for the least and the most educated males, respectively, while it peaked at 57 years of age among both the least and the most educated females (Figure 5A–C). The 95% CI of the age-specific mortality rates among both males and females overlapped at most ages, given a small number of subjects in each group.

**Figure 6:**
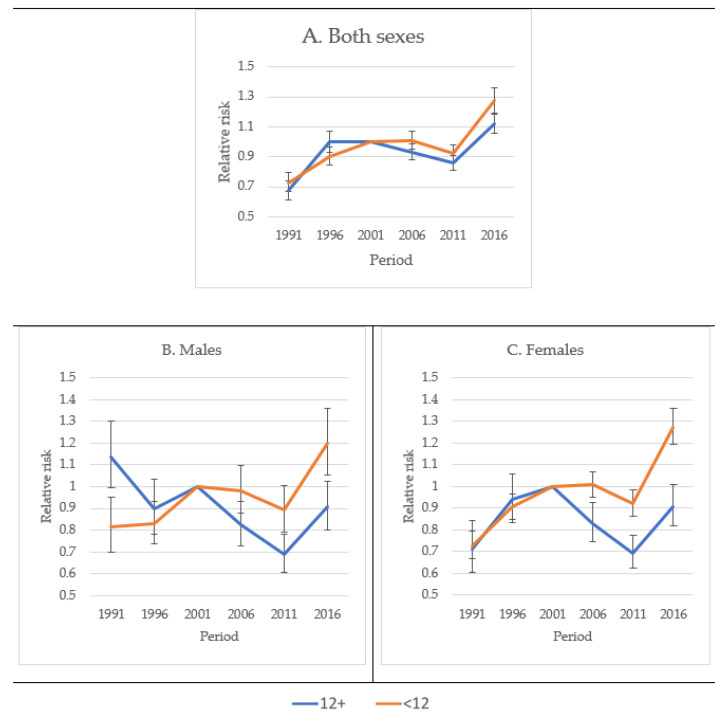
The relative risk of age-adjusted diabetes mortality among Hispanics by sex, year of death, and education level, USA 1989–2018. RR (Relative Risk) was compared to mortality in the year 2000.

APCWT compares the RR of dying to mortality at the midpoint of the studied period, in this case, 2001. The analyses showed an increased RR among both the least and the most educated members of both sexes together, as compared to mortality in 2001. The relative risk to mortality in 2001 was greater among the least educated than among the most educated after 2001. The difference in RR between the least and the most educated increased after 2001, indicating a higher risk of mortality for the former, compared to the latter, as demonstrated by the nonoverlapping confidence intervals after 2001 (Figure 6A–C).

**Figure 7:**
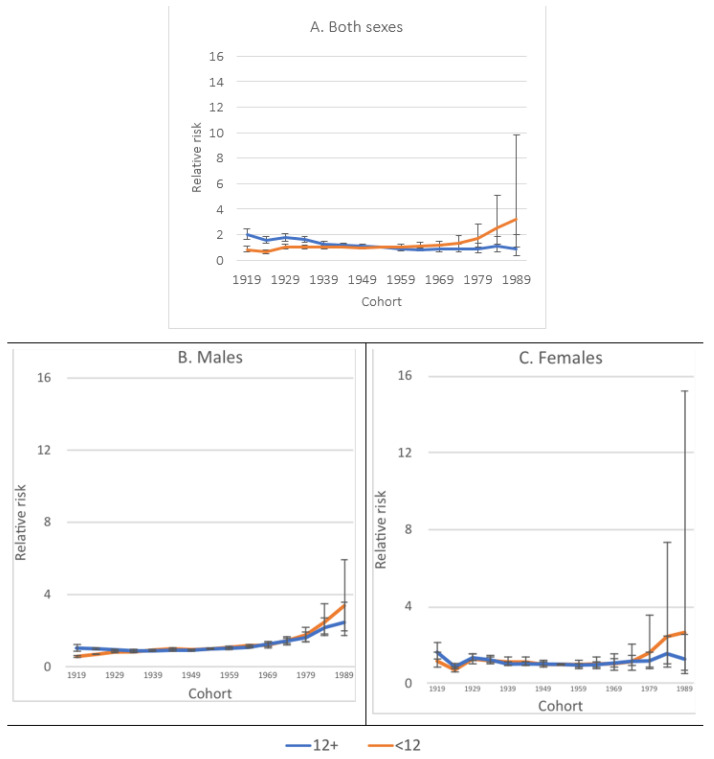
The relative risk of dying of diabetes among Hispanics in the USA by sex, birth cohort, and education level between 1989 to 2018. RR was compared to the 1954 cohort.

When compared to the mortality rates of the midpoint birth cohort (1954 in this case), a lower RR was observed among the most educated, compared to the least educated members of older cohorts, which was more visible among both sexes together and males. RR reversed among those born to cohorts after the 1954 cohort, showing increased rates among the least educated, compared to the most educated males and females, respectively. However, when comparing the RR among the least and the most educated for each group, we observed overlapping confidence intervals, indicating that the gap between RR for the least and the most educated among males and females, respectively, may be affected by a low number of observations in each group (Figure 7A–C).

## 4. Discussion

The results of the analyses undertaken in this study revealed that there are significant disparities in diabetes mortality between the least and most educated Hispanics in the USA. While the exact cause of this disparity remains unclear, the results of this study add to the existing literature by highlighting the role of educational attainment in predicting early mortality. Although the USA Hispanic population between 25 and 74 years of age doubled between 1989 and 2018, the number of diabetes-related deaths increased three times among members of this population segment during the same period. The results of this study showed a clear association between lower educational attainment, below 12th grade, and diabetes mortality among Hispanics. Overall, amenable mortality due to diabetes was higher among the most educated member of cohorts before 1954 than the least educated during the same period. Interestingly, this pattern reversed among members of younger cohorts after that year. This may imply that the most educated members of the cohorts before 1954 were more likely to live longer than the least educated from the same cohorts. The least educated member of cohorts before 1954 may have been affected by selective mortality, meaning they may have died before 1989 and therefore were not represented in this study. 

Hispanics in the USA are disproportionately affected by diabetes when compared to other race/ethnic groups as of 2017–2018, with Hispanic men reportedly more affected than Hispanic women [4]. The crude rate of diabetes mortality, as an underlying cause of death, in the USA general population was reported to be 25.7 per 100,000 inhabitants in 2017 [4], which is lower than the mortality rates among the least educated Hispanic men and women, as reported in the results of this study. Previous findings have highlighted a higher decrease in age- and sex-adjusted diabetes mortality rates among NHWs achieving a high school diploma than NHBs and Hispanics [24] attaining the same degree of education, suggesting that improving education may not benefit all segments of the USA population equally. Furthermore, the recent advancements in diabetes-related care in the USA [13] may not be equally available to the entire USA population, such as those from minority populations many of whom are among the most vulnerable. 

Gregg et al. estimated that the proportion of the Hispanic population with diabetes has increased more than twofold between 1985 and 2014. During the same period, the proportion of people with diabetes and less than a high school education had decreased from 45% to 23% of the total population with diabetes [2]. The authors also reported that the mortality among those with diabetes decreased by 12% and 3% among men and women, respectively, from 1988 to 2015 [2,12]. Between 1985 and 2011, years of life lost (YLL) to diabetes among Hispanics in the USA was estimated to outnumber all other races and ethnic groups [12], suggesting that Hispanics die at a younger age relative to members of other races or ethnic groups. 

The results of the analyses undertaken in this study showed that age-adjusted diabetes mortality rates among the least educated Hispanics were comparable to those reported for the least educated NHWs, but the age-adjusted diabetes mortality rates for the most educated Hispanics were notably higher than among the most educated NHWs [24]. Collectively, these findings suggest that Hispanics do not contribute in the same way as other races or ethnic groups to the observed decrease in diabetes mortality in the USA [2], in particular to NHWs. The protective effect of achieving a high school education seems to be stronger among the NHW than among the NHB and Hispanics in the United States. Consequently, it could be hypothesized that achieving high education may yield greater opportunities for increasing socioeconomic status (SES) and better health outcomes among NHWs than among NHBs and Hispanics.

The Hispanic population in the USA has increased from 50.7 million in 2019 to 60.6 million in 2020 and includes immigrants from more than 20 Latin American countries. However, as of 2018, 61% of the Hispanic population in the USA are of Mexican origin [41]. There is evidence that the prevalence of diabetes among USA Hispanics is heterogeneous and varies according to country of birth and other risk factors such as age, body mass index (BMI), physical activity, time spent in the USA, and other risk factors such as ethnic or racial background [6,7,10,42,43]. For example, a higher prevalence of diabetes has been observed in Hispanics living in the USA than among their peers living in their home countries [44]. The age-adjusted total prevalence of diabetes (diagnosed and undiagnosed diabetes) in 2011–2016 was comparable (19.3% to 24.6%) among Hispanics coming from different countries, except those coming from South America (12.3%), which is remarkably lower than other Hispanic groups such as those from Central America or the Caribbean. Furthermore, there is also a significant difference between the estimated proportion of Hispanics in the USA with diabetes achieving a high school education. The proportion of Hispanics not attaining a 12th-grade education has been shown to be 39%, but this proportion varies from 15% among those coming from South America to 55% among those coming from Central America from 2011 to 2016 [8]. Unfortunately, the lack of available data precluded our ability to undertake subanalyses to explore the relative contribution of these factors to the results reported herein, as the breakdown by country of origin of the number of deaths would result in a low number of cases in each group. Additionally, our objective was to determine the effect of not achieving a high school education on diabetes mortality among Hispanics in the USA as a group and not to demonstrate the difference of risk for diabetes mortality or for the risk of presenting with diabetes among Hispanics coming from different Latin American countries. 

There is evidence to suggest that various cardiovascular complications may already be present at the initial diagnosis of type 2 diabetes [45]. In such cases, these individuals would likely benefit from early preventive therapies, including lifestyle therapies, that may not be available to those with low education, such as minority groups in the USA. These complications can be the direct cause of death in many cases and to remove this potential bias from the analyses, only cases that had specified diabetes as the underlying cause of death were tabulated. Furthermore, the difference in mortality patterns reported herein does not account for exposure to poorer health behaviors. However, it can be assumed, based on previous research, that the least educated individuals are at a higher risk for obesity, poor nutrition, and lack of exercise [10,46], which are important risk factors for type 2 diabetes and diabetes control. 

As lower education is associated with reduced SES, income, and cultural differences, it is difficult to identify the relative importance of these effects on their own. Education in combination with occupation and income are the main components of SES, and this may be involved in the health disparity reported herein [47,48,49]. Furthermore, differences in diabetes mortality among USA cities have been demonstrated previously [3], with the highest diabetes mortality rates in El Paso, Texas, and the lowest in San Francisco, California. While interesting, this study did not aim to measure the effect of city variability on diabetes mortality among Hispanics, which would require the number of deaths and population estimates by age, sex, and education for the target population in each city, and therefore, these analyses were not undertaken.

In line with previous reports [2,12], the results of this study showed that the mortality rate increased with age for both men and women. However, the analysis of comparative age deviation suggested that age-adjusted mortality among males peaked at younger ages than among females, particularly among the least educated males for whom mortality peaked at 47 years of age, which is within productive age. 

This study has limitations that should be considered when interpreting the results of the analyses. Firstly, this is a retrospective study using data from various time points. Consequently, we were unable to determine whether increasing education through targeted interventions would lead to reduced diabetes mortality. Secondly, the lack of additional variables such as country of origin, time spent in the USA, BMI, and access to care preclude our ability to determine the relative importance of these variables on diabetes mortality in those with lower versus higher education. Furthermore, although we demonstrated that Hispanics in the USA with low educational attainment, as a group, have greater mortality than their well-educated peers, this study did not assess the ensuing consequences of low education. Finally, the variability in the number of death certificates with missing information about the level of education could potentially result in a selection bias. However, the proportion of Hispanic’s death certificates reporting diabetes as a cause of death, with unknown education, throughout the period of the study was only 5%. 

In summary, the results of this study demonstrated a powerful association between a low level of education and amenable mortality attributable to diabetes among Hispanics in the USA between 1989 and 2018. Importantly, higher diabetes mortality is but one of many inequalities affecting minorities with low educational attainment in the USA. Given the increasing prevalence of diabetes among the least educated Hispanics, which has also been reported elsewhere [50], there is an ever-growing need to identify and implement effective programs targeted to Hispanics with low educational attainment. Consequently, health policies in the USA should be implemented that focus on alleviating the growing health disparity in this subset of the population through targeted and tailored programs that can include preventative services, quality care, and self-management education to those most vulnerable, such as Hispanics with low educational attainment.

## Figures and Tables

**Table 1:** Number and crude mortality rates due to diabetes among Hispanics in the USA from 1989 to 2018.

	Education					
Sex/Period	<12 Grade		12+ Grade		All	
	N^o^ Deaths	Rate per 100,000	N^o^ Deaths	Rate per 100,000	N^o^ Deaths	Rate per 100,000
Males						
1989–1993	2348	18.39	1379	9.68	3727	13.80
1994–1998	3734	23.02	2722	13.85	6456	18.00
1999–2003	4492	23.10	3721	14.51	8213	18.21
2004–2008	5049	21.29	4682	13.57	9731	16.71
2009–2013	5177	21.29	5939	14.00	11,116	16.66
2014–2018	6269	32.18	8394	20.70	14,663	24.43
Females						
1989–1993	2887	22.39	1084	7.48	3971	14.50
1994–1998	4317	24.79	2183	10.86	6500	17.33
1999–2003	4791	26.16	2849	10.83	7640	17.13
2004–2008	4594	22.38	3233	9.48	7827	14.33
2009–1013	4288	20.17	3686	8.62	7974	12.46
2014–2018	4671	26.49	5269	12.48	9940	16.61
Both						
1989–1993	5235	20.4	2463	8.57	7698	14.15
1994–1998	8051	23.93	4905	12.34	12,956	17.66
1999–2003	9283	24.58	6570	12.65	15,853	17.67
2004–2008	9643	21.80	7915	11.54	17,558	15.56
2009–1013	9465	20.77	9625	11.30	19,090	14.60
2014–2018	10,940	29.47	13,663	16.51	24,603	20.52

## Data Availability

Data used for this analysis is available on request.

## Abbreviations

Name	Abbreviation
Non-Hispanic Whites	NHW
Non-Hispanic Blacks	NHB
United States of America	USA
95% Confidence Intervals	95%-CI
Probability	p
Age–Period–Cohort Model	APCM
Age–Period–Cohort Web Tool	APCWT
Average Annual Percent Change	AAPC
Body Mass Index	BMI
Centers for Disease Control and Prevention	CDC
Relative Risk	RR
